# Protective potential of fresh orange juice against zinc oxide nanoparticles‐induced trans‐placental and trans‐generational toxicity in mice

**DOI:** 10.1002/fsn3.3470

**Published:** 2023-06-06

**Authors:** Chaman Ara, Shagufta Andleeb, Shaukat Ali, Barirah Majeed, Asia Iqbal, Madeeha Arshad, Asma Chaudhary, Asmatullah, Aliza Muzamil

**Affiliations:** ^1^ Institute of Zoology University of Punjab Lahore Pakistan; ^2^ Division of Science and Technology, Department of Zoology University of Education Lahore Pakistan; ^3^ Applied Entomology and Medical Toxicology Laboratory, Department of Zoology Government College University Lahore Pakistan; ^4^ Department of Wildlife and Ecology University of Veterinary and Animal Sciences Lahore Pakistan

**Keywords:** multigenerational, orange juice, toxicity, trans‐placental, zinc oxide nanoparticles

## Abstract

Due to the emerging applications of nanoparticles, human exposure to nanoparticles is unavoidable, particularly to zinc oxide nanoparticles (ZnO NPs), owing to their wide range of usage. The ongoing study aimed to evaluate trans‐generational toxic potential of ZnO NPs through exposure to F0 mothers, in F1 pups and F1 mature offspring and the protective potential of fresh orange juice (OJ). Twenty‐eight F0 mothers were randomly allocated into four groups (*n* = 7), control; untreated, dose group; exposed to ZnO NPs, dose+antidote group; coadministered ZnO NPs + OJ, antidote group; OJ, during the organogenetic period. Fifty percent of F0 mothers were subjected to cesarean sections on the 18^th^ day of gestation and F1 pups were recovered, macro‐photographed, and dissected for liver evisceration, while 50% of F0 mothers underwent standard delivery. After parturition, F1 offspring were examined, and the liver and blood samples were extracted. Observations showed that ZnO NPs exposure in F0 mothers in preparturition and postparturition resulted in decreased body weight, increased liver weight, and elevated levels of ALT and AST significantly *p* ≤ .05 as compared to the control and antidote groups. Histopathological analysis of maternal livers intoxicated with NPs showed the disruptive structure of central vein, hepatocytes, and Kupffer cells in F0 mothers, while F1 pups showed morphological deviations and distorted development of the liver tissue and congestion, in contrast to the control. F1 offspring of NPs exposed mothers, even at postnatal week 8 showed pyknotic nuclei and activated Kupffer cells in the liver sections against control. But in the case of the Dose+antidote group, alterations were less severe than in the dose group. It can be concluded that exposure to ZnO NPs instigates teratogenicity and hepatotoxicity in F1 pups, F0 mothers, and F1 offspring, respectively, while fresh orange juice acts as a remedial agent against the abovementioned toxicities.

## Graphical abstract

Evaluation of the trans‐generational toxic potential of Zinc Oxide nanoparticles (ZnO NPs) through exposure to F0 mothers, in F1 Pups and F1 mature offspring and the protective potential of fresh orange juice (OJ) against the probable toxicity

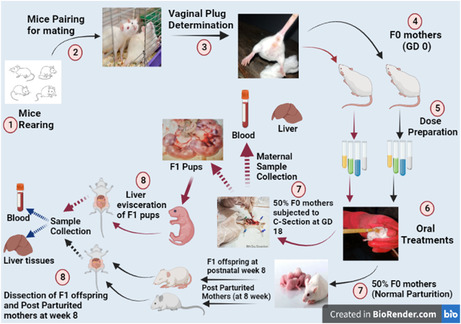

## 1 INTRODUCTION

Nanotechnologies are now making influential progress in different domains of human lives. Metal oxide nanoparticles are used as a substitute for trace elements and bulk particles, due to their unique properties and diverse nanostructure, and are widely used in ceramics, cosmetics, pigments, and the food industry as an additive and for other business purposes (Teschke & Eickhoff, 2016; Dhapte et al., 2015). Nanoparticles (NPs) have specific chemical and physical properties due to their high surface area and nanoscale size (Khan et al., 2019). Their applications, durability, and some other qualities also depend on their specific size, configuration, and composition. Due to all these features, they become the best materials for numerous military and commercial applications, including catalytic activity, visualization, therapeutic diagnosis, energy‐based research, and ecological applications as well. Heavy metal NPs of lead, mercury, and tin are known to be even more compact and stable so their deterioration could not be easily attained, which might result in bioaccumulation and several toxic effects (Khan et al., 2019).

Zinc is an important trace element, which constitutes most of the essential enzymes in humans. Currently, ZnO NPs are also used as a Zn nutritional supplement for food. Among bio‐metallic particles, zinc oxide nanoparticles are of greater importance due to their excellent antimicrobial activities and radiation protective abilities owing to being an integral part of commercially available edible products, pharmaceuticals, and cosmetics and subsequently, present in marine and terrestrial habitats, which causes toxicity in organisms by interfering with normal physiological mechanisms of embryos, growing animals, and adults (Abbasalipourkabir et al., 2015; Exbrayat et al., 2015; Waseem & Divya, 2020).

ZnO NPs induced toxicity has been recorded in the reproductive system of pregnant females leading to teratogenic and trans‐generational abnormalities as well. ZnO NPs may enter the body of pregnant females through breathing, intravenous injection, ingestion, or permeation of the skin, which activates infections, reactive oxygen species (ROS), apoptosis, and dyscrasia in the reproductive system. Some reports indicated that ZnO NPs disorganize the structure of oocytes by penetrating the boundaries of zona pellucida, theca cells, and granulose cells (Zhai et al., 2018). They also disturb sex hormones by activating different secretory cells (Jo et al., 2013; Keerthana & Kumar, 2020). NPs cross placental barriers and may cause genotoxicity, and disrupt developmental processes, which cause developmental anomalies, weight gain, and poor fetal immunity (Hong et al., 2014; Singh 2019). The intensity of toxic effects depends upon their concentration, size, and charge on it (Hou & Zhu, 2017).

Due to their nano size, they can absorb through the skin or from the gut to blood circulation and reach other organs. The liver, spleen, heart, pancreas, and bone are targeted organs for ZnO NPs toxicity (Abbasalipourkabir et al., 2015; Hong et al., 2013; Wang et al., 2008). As the liver is performing a pivotal role in the detoxification of unneeded materials, so it is the most vulnerable organ to instigated toxicity. Some researchers reported that a substantial deposition of nanoparticles in the liver results in tissue damage. The mechanism behind the ZnO NPs toxicity is that the overproduction of ROS creates an oxidant–antioxidant imbalance, which leads to the disruption of tissues. Disturbance in the normal redox status of tissues can lead to DNA damage, irregular cell signaling, apoptosis, and change in cell motility (Fu et al., 2014; Yousef et al., 2019). Another study reported that ZnO nanoparticles have also been expected to induce oxidative tissue damage by increasing lipid peroxidation (Sharma, Anderson, & Dhawan, 2012; Sharma, Singh, et al., 2012).

On the other hand, citrus juices are considered a prime source of phytochemicals, having a substantial amount of polyphenols, flavonoids, carotenoids, minerals, and vitamins. Currently, polyphenolic compounds are trendy in medicine and food products due to their potential health benefits (Selamoglu, 2018; Dhakal & Sharma, 2020). All these bioactive compounds have pharmacological activities as potent radical scavengers, so can protect against ROS‐induced toxicities (Rolle et al., 2016; Youness et al., 2016). Many studies inferred that polyphenols and flavonoids in fruits have potent roles in the prevention and treatment of various health issues. Studies showed that flavonoid‐rich foods reduced the risk of developing cancer (Selamoglu, 2017a, 2017b).

The literature quoted above shows that ZnO NPs cause toxicity in various systems and models but the results are still inconclusive. According to our knowledge, trans‐placental as well as trans‐generational toxicity specifically, teratogenicity and multigenerational hepatotoxicity are not reported yet. Besides, the protective potential of orange juice against ZnO NPs instigated embryo and hepatotoxicity remains obscure. So, it is inevitable to conduct a multiparametric study to follow up aforementioned toxicities and protection with orange juice. Working on the mammalian model results can be extrapolated to humans. Based on these findings, a thorough risk evaluation will be ensured for newly designed nanoparticles before launch in commercial retails.

## 2 MATERIALS AND METHODS

### 2.1 Ethics statement

This experiment was performed on Swiss Albino mice *Mus musculus* at the Animal House of The Institute of Zoology, University of the Punjab, Lahore, following ethical considerations by the Institutional Ethics Review Board (which can provide a certificate on‐demand) on animal experimentation as well as international standards. The nearby way is the Wet op de dierproeven (article 9) of Dutch law (international) and an associated rule planned via the Bureau of Animal Research Licensing, as reported in our previous articles (Ali et al., 2019; Ara, Asmatullah, Butt, et al., 2021, Ara, Asmatullah, Yaseen, et al., 2021; Khan et al., 2019). This article does not contain any studies with human participants performed by any of the authors.

### 2.2 Animal rearing

Mice (12 females and 6 males) 6–8 weeks old and having body weight 23 ± 2 g were purchased from Veterinary Research Institute (VRI), Lahore. All animals were reared in steel cages, under standard conditions of 12:12 light/dark cycle at 28 ± 2°C room temperature with 40%–60% humidity. All of them are provided with distilled water and food ad libitum, feed no 12, National Feed industries private Ltd. All mice were acclimatized for 1–2 weeks before mating. After 1 week, the colony was raised by keeping one male with two females in a separate cage. After raising the colony, the mother along with the pups was placed in separate cages for up to 4 weeks and then the pups were weaned. This progeny of mice was used in the experiment.

### 2.3 Determination of vaginal plug

During the estrous phase, two female mice were paired with one male mouse. A vaginal smear was checked regularly early in the morning as a signal of successful mating. The appearance of a vaginal plug on a particular day is considered gestation day 0 (GD 0). Based on the mating plug, females were placed in separate cages and marked accordingly. These females were considered pregnant mothers “F0”, observed, and weighed routinely from gestation days 0–20.

### 2.4 Chemicals

Zinc oxide NPs were formulated by using Green synthesis (for preparation details, visit our article, Iqbal et al., 2021). Enzyme assay kits were purchased from Bio Vision. Formalin, sodium carbonate, ferrous sulfate, Folin–Ciocalteu reagent were used from Merck. Gallic acid, 2, 4, 6‐tripyridyl‐triazine, (TPTZ), hematoxylin and eosin stains (H&E), and ethanol were purchased from Sigma Aldrich. Isoflurane was purchased from Bimeda. All chemicals used during this work were of analytical grades.

### 2.5 Dose preparation and administration

*Allium cepa*‐based zinc oxide nanoparticles of size 50–90 nm were prepared through green synthesis. The procedure for preparation as well as the complete characterization of NPs is described in our previous publication (Iqbal et al., 2021; Vinay & Chandrasekhar., 2021). Zinc 14–20 mg/kg/BW is approved by World Health Organization as an acceptable daily intake (ADI) in adults. The NPs dose used was 25 μg/g/BW and the solution was prepared in distilled water so that 0.1 mL of solution contained the desired dose of ZnO NPs. Treatments were given via oral gavage from gestation days 6–12 with the help of a glass syringe, coupled with a blunt rubber tube.

### 2.6 Orange juice preparation

Fresh oranges were shopped, washed, and peeled thoroughly. Oranges were squeezed to get fresh orange juice (OJ). Dilution of concentrated orange juice was made by adding 1 mL of water to 1 mL of orange juice. Aqueous orange juice (2 mL) was orally given to pregnant F0 mothers from the 6th to 12th day of gestation with and without NPs.

### 2.7 Experimental design

**FIGURE 1:**
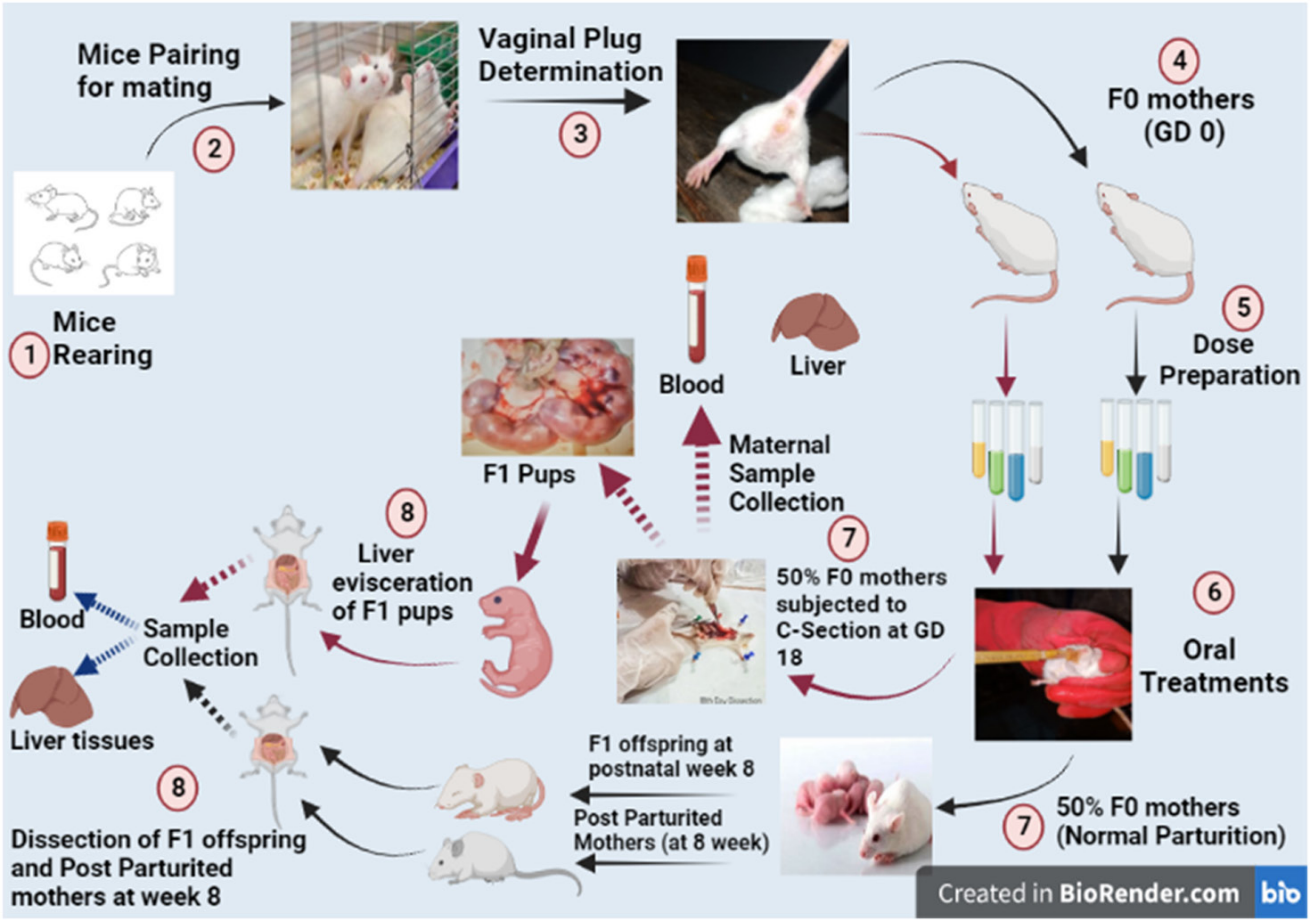
Scheme of sorting, dosing, and sampling in the experiment.

In total, 28 pregnant F0 mothers (mice) were segregated randomly into four groups (*n* = 7): control (provided with regular food and water ad libitum), dose Group (treated with 25 μg/kg/BW ZnO NPs), the antidote group (exposed to 2 mL of dilute fresh OJ), and dose + antidote group (treated with 25 μg/kg/BW ZnO NPs + 2 mL of diluted fresh OJ). All treatments were given forenoon, once a day from GD 6th to 12th. Maternal body weight was monitored routinely. Fifty percent of pregnant females were subjected to C‐section at day 18 of gestation, after 5% isoflurane anesthesia. Maternal liver, F1 pups' liver, and blood were recovered for histopathological and biochemical analysis, respectively. The rest of 50% of pregnant females were subjected to normal delivery. After birth, offspring were kept with their mothers under a controlled environment till postnatal week 4 and then weaned. At postnatal week 8, mothers (undergo parturition) and their offspring were euthanized. The liver tissues and blood samples were (*p* < .05) recovered for further analysis (Figure 1).

### 2.8 Morphological and morphometric observations

After euthanization of F0 mothers, F1 pups, and F1 offspring, the liver tissues were recovered. The liver tissues were kept in 10% formalin solution overnight and transferred to 70% ethanol for morphological observations. Tissues were observed under a binocular microscope and digital images were captured using a digital camera. Measurement of the liver weights was done using the weighing machine.

### 2.9 Biochemical parameters

Blood sample collection was done through cardiac puncture with 2‐mL sterilized syringes under deep anesthesia. Samples were stored in clotted vials, Serum was separated by centrifuging blood samples and stored at −80°C till assayed. Serum was examined for the liver function tests (LFTs) by using Bio Vision company kits of high sensitivity for the quantification of the liver key markers.

### 2.10 Histopathological study

The liver tissues were fixed in 10% formalin for 24 h, and then shifted to 70% ethanol. Dehydration was done in increasing concentrations of ethanol (70%, 80%, 90%, and 100%) and kept in 100% ethanol overnight with gentle shaking. Samples were cleaned with xylene before embedding and transferred to paraffin wax overnight at 58 ± 2°C to infuse wax in the liver tissues. After wax infiltration, the blocks were prepared by pouring molten wax into the specially designed glass cavities and tissue was placed in them. Air bubbles, if present, were removed with the help of a red‐hot needle. After solidification, the wax was trimmed in a cube shape to make a block and fix on a wooden piece with the help of molten wax as a binding agent. Five‐micrometer‐thick tissue section was taken by semiautomatic microtome. Wax ribbons containing tissue sections were transferred to a warm water bath and shifted on slides. Sections were deparaffinized with xylene and rehydrated with alcohol and water. After rehydration, sections were stained with H&E stains and allowed to dry in a dust‐free environment (for more details, see our previous articles [Asmatullah et al., 2018]). Slides were examined under a microscope at 40 and 100× for further histopathological analysis.

### 2.11 Estimation of the antioxidant activity (FRAP assay)

The antioxidant activity of the liver tissues and fresh orange juice was evaluated using FRAP (ferric reducing antioxidant power) assay. The basic principle of this method is to convert the ferric‐triazine complex into ferrous, color changes in the presence of antioxidants. To evaluate the FRAP assay, chilled KCl buffer solution 1–2 mL was added to 100 mg of the liver tissues. The samples were homogenized with a homogenizer and centrifuged at 19,118 *g* for 7 min at temp. 4°C. After centrifugation, 150 μL of supernatant was shifted into labeled Eppendorf by using a micropipette. FRAP reagent contained 100 mL of acetate buffer added in 10 mL of freshly prepared TPTZ with 10 mL of FeCl_3_ solution and kept at 37°C. To prepare a stock solution, 27.8 mg of ferrous sulfate was dissolved in 100‐mL distilled water by shaking it gently. Serial dilutions of standard solutions were prepared through the addition of 0, 2, 4, 6, 8, and 10 mL of the above‐prepared stock solution in the conical flasks maintaining volume at 100 mL by adding distilled water. FRAP reagent was used as blank. Aliquots of 150 μL of sample supernatant were mixed with distilled water along with 2850‐μL FRAP reagent. The optical density of the mixture was measured spectrophotometrically at 593 nm. Results were expressed as ascorbic acid equivalents, μmol ascorbic acid/100 mL. A standard curve was plotted in the case of orange juice and bar graphs were drawn with FRAP values of the liver homogenates in respective groups.

### 2.12 Quantification of total phenolic content

The total phenolic content (TPC) of fresh orange juice was analyzed to evaluate its antioxidant content by Folin–Ciocalteu assay following the procedure set by Franco et al. (2002) and Asami et al. (2003). Fifty‐microliter fresh orange juice was mixed with 100 μL of Folin–Ciocalteu reagent. After 8 min, 300 μL of Na_2_CO_3_ was added to the mixture with 1.58 mL of distilled water and mixed thoroughly. The mixture was kept at 40°C for 30 min after which absorbance was measured at 593 nm. TPC was represented as gallic acid equivalents per 100 mL of the orange juice (mg gallic acid/100 mL of orange juice).

### 2.13 Statistical analysis

All data were represented in terms of mean ± SEM for each group. SPSS Version 20 was used for statistical evaluation. Significant difference among multiple groups was evaluated through one‐way ANOVA. The lowest significant difference *p* < .05 was considered statistically significant.

## 3 RESULTS

### 3.1 Morphological analysis

#### 3.1.1 Maternal body weight before parturition (till GD 18)

**FIGURE 2:**
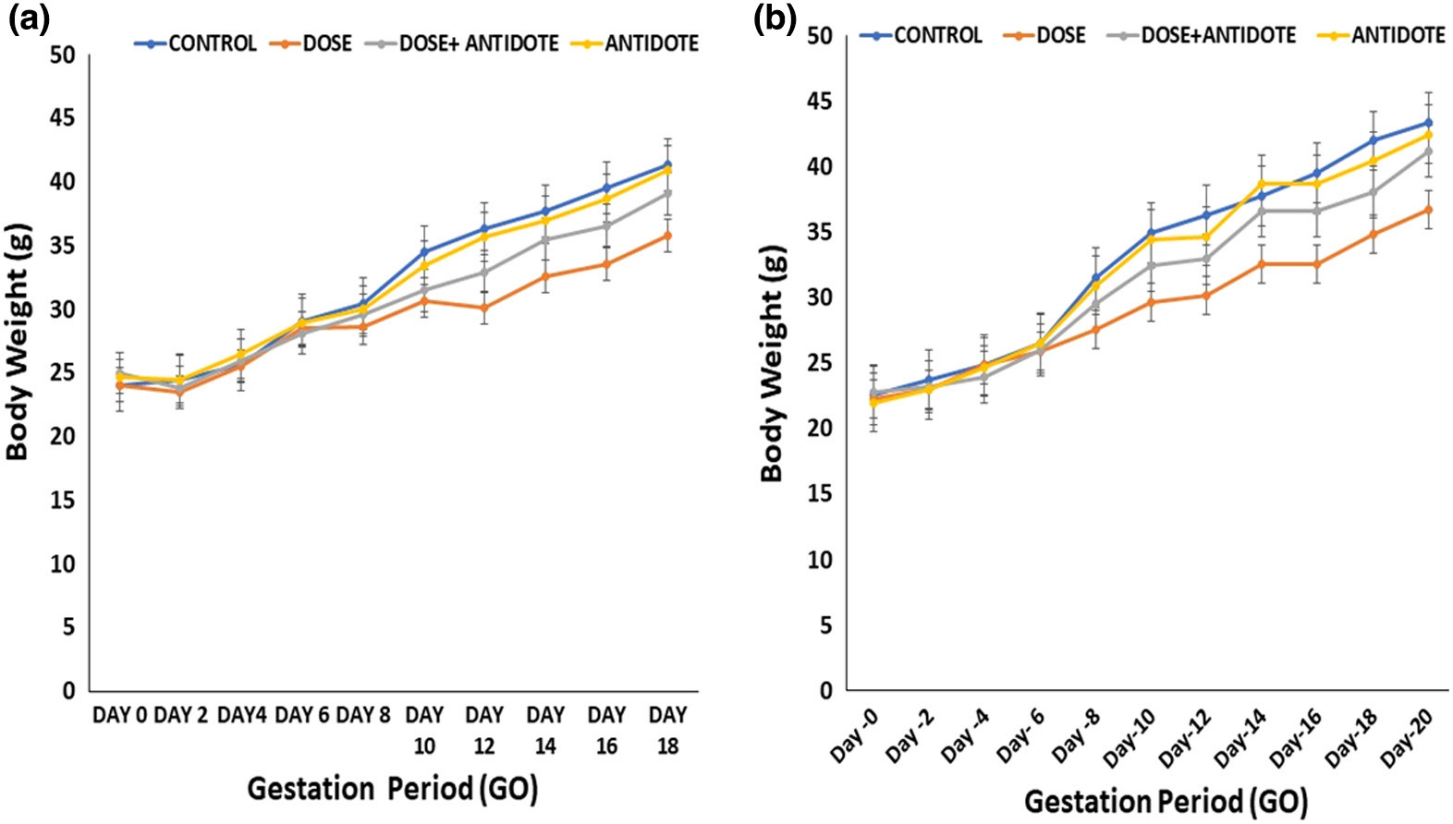
(a) Body weight of F0 mothers before parturition. (b) Body weight of F0 mothers till parturition. Graphs depict a remarkable decrease in weight gain ratio in the dose group against the control while maternal body weight in dose + antidote group remains incomparable range with control.

Maternal body weight was evaluated from day 0 to day 20 of the gestational period before parturition. Mice treated with ZnO NPs showed less gain in body weight in comparison with the control (*p* < .01). The maternal body weight gain in dose+antidote (ZnO NPs + OJ) and antidote (OJ) groups was significantly higher than the dose group and compared with the control group as shown in Figure 2a.

#### 3.1.2 Maternal body weight till parturition

Maternal body weight was evaluated until the 20th day of the gestational period just before parturition. The body weight of F0 mothers in the control group increased significantly (*p* < .05) from the initial weight. A slight reduction in weight gain in body weight of F0 mothers in the dose + antidote group (ZnO NPs + Fresh OJ) and the antidote group (fresh OJ) was observed at GD 14 in comparison with control *p* < .01 which was restored at GD 20. The group treated with ZnO NPs showed a decrease of *p* < .05 in maternal weight gain when compared with the control as shown in Figure 2b.

### 3.2 Organ weight measurements

#### 3.2.1 Maternal liver weight at preparturition

**FIGURE 3:**
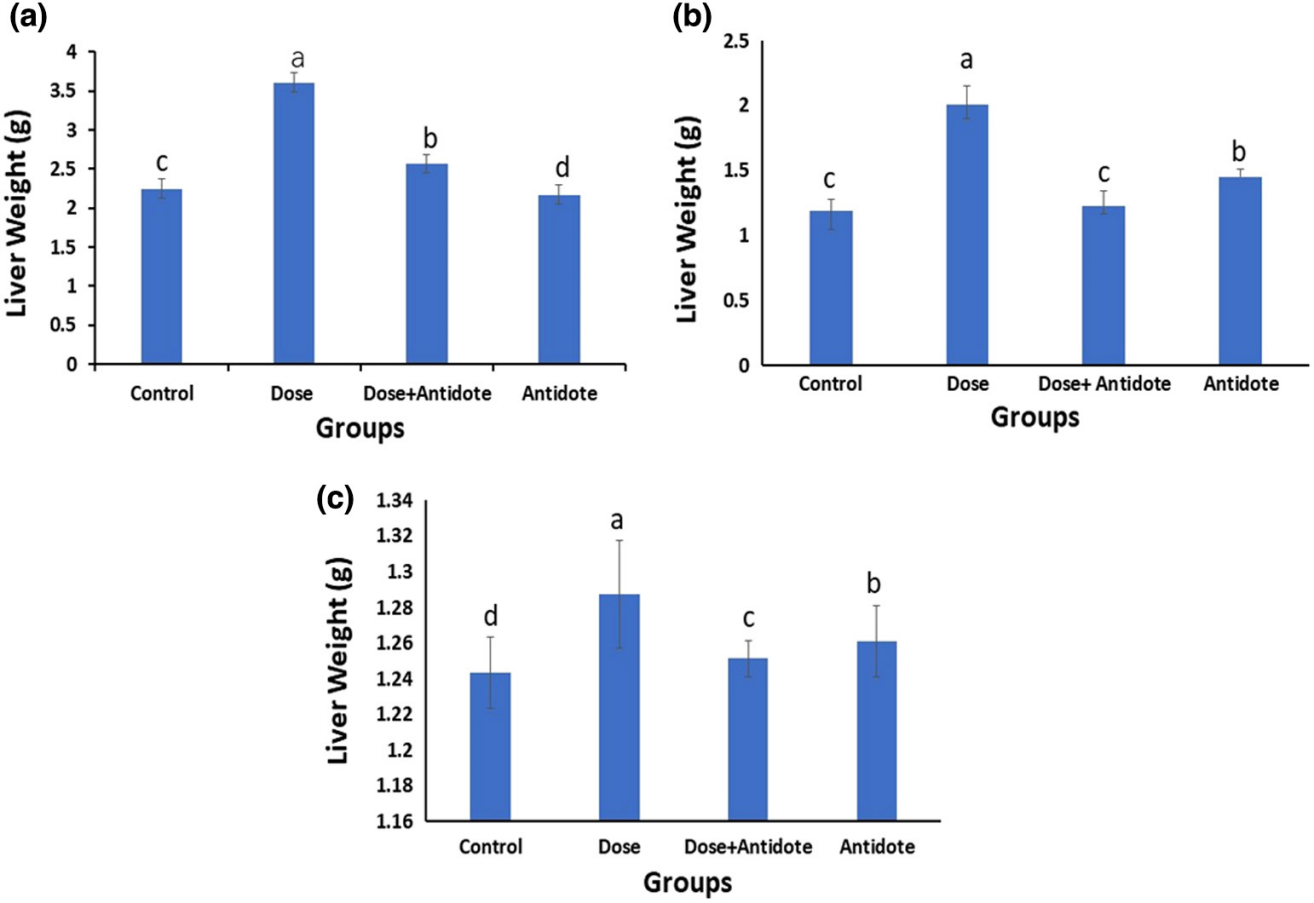
(a) Maternal liver weight at preparturition. (b) Maternal liver weight at postparturition week 8. (c) The liver weight of F1 mice at postnatal week 8. Bar graphs depict that the liver weight in ZnO NPs treated group increased significantly by *p* < .05 in F0 mothers and F1 offspring as compared to the control of the same cadre. Values represented are in terms of mean ± SEM.

The average weight of the maternal liver in the control group on the 18th day of gestation was 2.25 ± 0.12 g. ZnO NPs exposed F0 mothers showed a significant increase in the liver average weight (3.61 ± 0.39 g) as compared to control (*p* < .05). In the dose+antidote group, the increase was less obvious but the antidote group did not show an increase in the liver weight as compared to control as shown in Figure 3a.

#### 3.2.2 Maternal liver weight at postparturition week 8

Maternal liver weight was remarkably high in ZnO NPs treated mothers (2.01 ± 0.14 g) at postparturition week 8 as compared to control group mothers (1.19 ± 0.09 g). The average liver weight in dose+antidote and antidote groups was almost similar to the control as shown in Figure 3b.

#### 3.2.3 The liver weight of F1 mature offspring at postnatal week 8

The average liver weight of F1 mice in the dose group showed a noteworthy increase (1.29 g ± 16.23) in the eighth week of the postnatal period as compared to the control (1.24 g ± 9.12) but less obvious than in directly exposed individuals. A slight increase in the liver weight was observed in the antidote group too against the control group, as shown in Figure 3c.

### 3.3 Biochemical analysis

#### 3.3.1 Maternal LFT at preparturition

The total bilirubin, aspartate transaminase (AST), alanine aminotransferase (ALT), alkaline phosphatase (ALP), and total protein levels in serum were measured in all groups at day 18 of gestation. The ALT, AST, and ALP (69.0 ± 0.72, 89.2 ± 0.14, and 290.6 ± 7.26) levels significantly increased in the dose group as compared to the control (45.6 ± 0.87, 57.0 ± 0.10, and 228.1 ± 10.2) *p* < .05, whereas ALT (57.6 ± 0.67) and AST (58.4 ± 0.11) levels were in the normal range in dose + antidote group compared to the control group. The total bilirubin, direct bilirubin, and indirect bilirubin (0.60 ± 0.44, 0.2 ± 0.011, 0.42 ± 0.26) showed a significant decrease *p* < .05 in the dose group in comparison with the control (0.86 ± 0.30, 0.36 ± 0.015, and 0.5 ± 0.31) but stayed in range.

The total protein level and A/G ratio% showed nonsignificant results in all groups except in the dose group where these parameters significantly deviated *p* < .05 from the control group, justifying biometric observations of the maternal liver (Table 1).

**TABLE 1:** Functional key markers of the liver in F0 mothers at preparturition.

Groups	Control	Dose	Antidote	Dose + Antidote
Parameters	*n* = 7	*n* = 7	*n* = 7	*n* = 7
Total bilirubin (mg/dL)	0.86^c^ ± 0.30	0.60^a^ ± 0.44	0.77^bc^ ± 0.21	0.66^b^ ± 0.33
Bilirubin direct (mg/dL)	0.36^b^ ± 0.015	0.27^a^ ± 0.011	0.34^b^ ± 0.016	0.29^a^ ± 0.013
Bilirubin indirect (mg/dL)	0.51^c^ ± 0.31	0.42^a^ ± 0.26	0.48^bc^ ± 0.30	0.45^b^ ± 0.29
ALT (U/L)	45.6^a^ ± 0.87	69.0^c^ ± 0.72	48.7^a^ ± 0.95	57.6^b^ ± 0.67
AST (U/L)	57.0^a^ ± 0.10	89.2^b^ ± 0.14	57.3^a^ ± 0.13	58.4^a^ ± 0.11
ALP (U/L)	228.1^a^ ± 10.2	290.6^b^ ± 7.26	235.6^a^ ± 9.90	282.0^b^ ± 8.43
Total protein (g/dL)	8.23^a^ ± 0.70	10.12^b^ ± 0.42	8.09^a^ ± 0.55	8.34^a^± 0.65
Albumin (g/dL)	4.29^ab^ ± 0.16	4.55^a^ ± 0.16	4.65^b^ ± 0.17	4.34^a^ ± 0.14
Globulins (g/dL)	4.10^a^ ± 0.12	5.64^b^ ± 0.08	4.37^a^ ± 0.10	3.82^a^ ± 0.10
A/G ratio (%)	1.46^b^ ± 0.03	0.81^a^ ± 0.01	1.35^b^± 0.03	1.08^b^ ± 0.02

*Note*: All values are represented as Mean ± SE. Different alphabets in rows show the significant difference between groups.

Abbreviation: *n*, number of samples.

#### 3.3.2 Maternal liver function test at postparturition week 8

The ALT, AST, and ALP increased significantly by *p* < .05 in dose (101 ± 0.15, 88.0 ± 0.84, and 143.6 ± 0.14) and dose + antidote (91.2 ± 0.13, 79.0 ± 0.68, and 109.3 ± 0.15) groups as compared to the control (85.4 ± 0.16, 59.0 ± 0.87, and 112.5 ± 0.17) but in the group, which is cotreated with NPs and OJ variations in enzyme level were less severe than solely NPs treated mothers. While the total bilirubin level showed a significant decrease in the dose group and dose + antidote group, the antidote group showed nonsignificant results against control. The total protein level observed in the NPs exposed was notably high; conversely, A/G ratio% was lower than the control as shown in Table 2.

**TABLE 2:** Enzymatic key markers of the liver in F0 mothers at postparturition.

Groups	Control	Dose	Antidote	Dose + Antidote
Parameters	*n* = 7	*n* = 7	*n* = 7	*n* = 7
Total bilirubin (mg/dL)	2.70^b^ ± 0.29	2.31^a^ ± 0.34	2.53^b^ ± 0.52	2.37^a^ ± 0.42
Bilirubin direct (mg/dL)	0.61^b^ ± 0.01	0.49^a^ ± 0.03	0.57^b^ ± 0.03	0.46^a^ ± 0.02
Bilirubin indirect (mg/dL)	2.07^b^ ± 0.03	1.83^a^ ± 0.03	1.87^a^ ± 0.02	1.98^ab^ ± 0.03
ALT (U/L)	85.4^ab^ ± 0.16	101^c^ ± 0.15	73.5^a^ ± 0.14	91.2^b^ ± 0.13
AST (U/L)	59.0^a^ ± 0.87	88.0^b^ ± 0.84	64.4^a^ ± 0.79	79.0^ab^ ± 0.68
ALP (U/L)	112.5^b^ ± 0.17	143.6^c^ ± 0.14	86.3^a^ ± 0.16	109.3^b^ ± 0.15
Total Protein (g/dL)	7.13^b^ ± 0.10	8.17^c^ ± 0.06	7.25^b^ ± 0.13	6.67^a^ ± 0.12
Albumin (g/dL)	4.09^b^ ± 0.017	3.75^a^ ± 0.011	4.32^c^ ± 0.092	3.93^b^ ± 0.81
Globulin (g/dL)	3.07^c^ ± 1.06	4.12^a^ ± 0.06	3.72^c^ ± 0.73	3.02^b^ ± 0.13
A/G ratio (%)	1.34^b^ ± 0.07	0.84^a^ ± 0.04	1.47^b^ ± 0.85	2.25^c^ ± 0.74

*Note*: All values are represented as Mean ± SE. Different alphabets in rows show the significant difference between groups.

Abbreviation: *n*, number of samples.

#### 3.3.3 The liver function test analysis of F1 mice at postnatal week 8

The liver enzyme analysis in F1 mice showed an insignificant increase in levels of ALT, AST, and ALP in the dose group (101.4 ± 0.90, 137.5 ± 0.23, and 295.8 ± 2.96) and dose + antidote group (91.2 ± 0.84, 148.6 ± 0.29, and 227.5 ± 3.94) as compared to control (89.4 ± 0.79, 119.5 ± 0.26, and 245.4 ± 3.34). This shows that the effects of ZnO NPs induced biochemical alterations diluted in the next generation to a great extent, while total bilirubin levels decrease significantly in the dose group (1.13 ± 0.13) in comparison with control (2.10 ± 0.16) but remain in the reference range. Total protein levels and A/G ratio % showed little bit deviations in NPs exposed groups in comparison with control but values fall in the normal range, as shown in Table 3.

**TABLE 3:** Functional key markers of the liver in F1 offspring at postnatal week 8.

Groups	Control	Dose	Antidote	Dose + Antidote
Parameters	*n* = 7	*n* = 7	*n* = 7	*n* = 7
Bilirubin total (mg/dL)	2.10^c^ ± 0.16	1.13^a^ ± 0.13	2.25^d^ ± 0.14	1.75^b^ ± 0.16
Bilirubin direct (mg/dL)	0.65^b^ ± 0.02	0.48^a^ ± 0.02	0.69^c^ ± 0.01	0.62^b^ ± 0.01
Bilirubin indirect (mg/dL)	1.45^c^ ± 0.01	0.65^a^ ± 0.02	1.55^d^ ± 0.02	1.15^b^ ± 0.01
ALT (U/L)	89.4^a^ ± 0.79	101.4^b^ ± 0.90	99.8^a^ ± 0.87	91.2^a^ ± 0.84
AST (U/L)	119.5^a^ ± 0.26	137.5^b^ ± 0.23	91.3^a^ ± 0.25	148.6^c^ ± 0.29
ALP (U/L)	245.4^a^ ± 3.34	295.8^c^ ± 2.96	265.5^b^ ± 3.65	237.5^a^ ± 3.94
Total protein (g/dL)	7.16^c^ ± 0.18	5.25^a^ ± 0.17	6.50^b^ ± 0.16	5.67^a^ ± 0.18
Albumin (g/dL)	4.35^c^ ± 0.12	3.37^a^ ± 0.13	3.95^b^ ± 0.11	3.55^a^ ± 0.11
Globulin (g/dL)	2.75^c^ ± 0.06	1.45^a^ ± 0.05	2.25^b^ ± 0.07	1.74^a^ ± 0.05
A/G ratio (%)	1.54^a^ ± 0.04	1.79^b^ ± 0.04	1.55^a^ ± 0.05	1.67^a^ ± 0.03

*Note*: The values are represented as Mean ± SEM. Different alphabets in rows show the significant difference between groups.

Abbreviation: *n*, number of samples.

### 3.4 Morphological analysis

**FIGURE 4:**
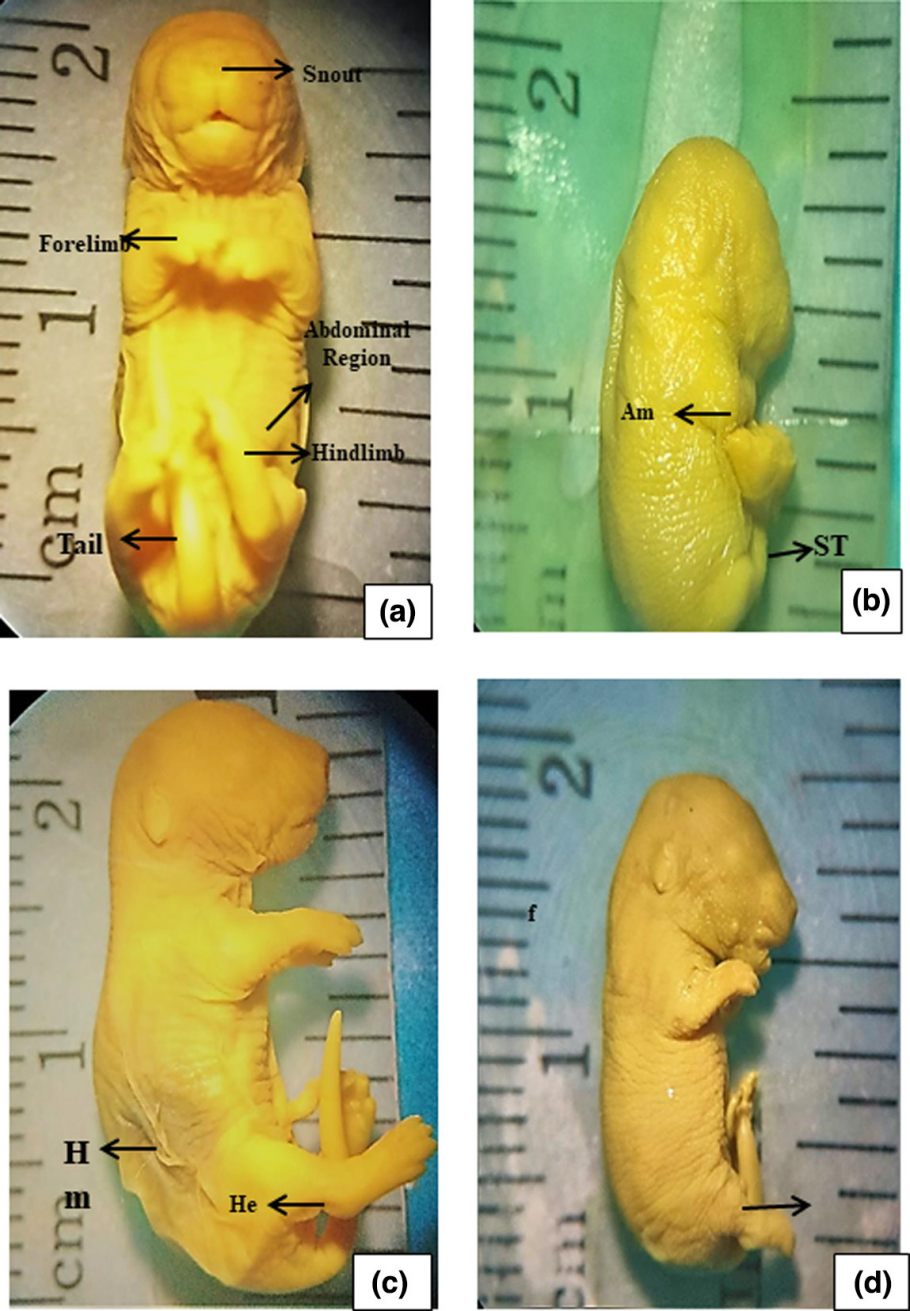
Photographs of F1 pups from F0 mothers recovered at day 18 of gestation (a) control group showing regular morphological features, tail, snout fore, and hind limbs. (b) ZnO NPs treated group showing morphological anomalies like Am, Amelia; ST, short tail, (c) dose + antidote: He, hyper‐extension; Hm, hemorrhage and (d) antidote group, dl, drooped leg.

Morphological analysis of recovered fetuses was done to observe abnormalities in treated groups. The fetus recovered from the mother on day 18th of gestation and showed normal physical structures. The control group fetuses showed normal snout, fore as well as hind limbs, and other craniofacial features. Dose‐treated group showed Amelia, a short tail, while the dose + antidote group showed minor defects like seldom hemorrhage and hyper‐extension of limbs. Antidote‐treated group revealed a drooped leg in one embryo while all other embryos were normal as shown in Figure 4.

### 3.5 Histopathological observations of the liver

#### 3.5.1 Microanatomy of maternal liver at preparturition

**FIGURE 5:**
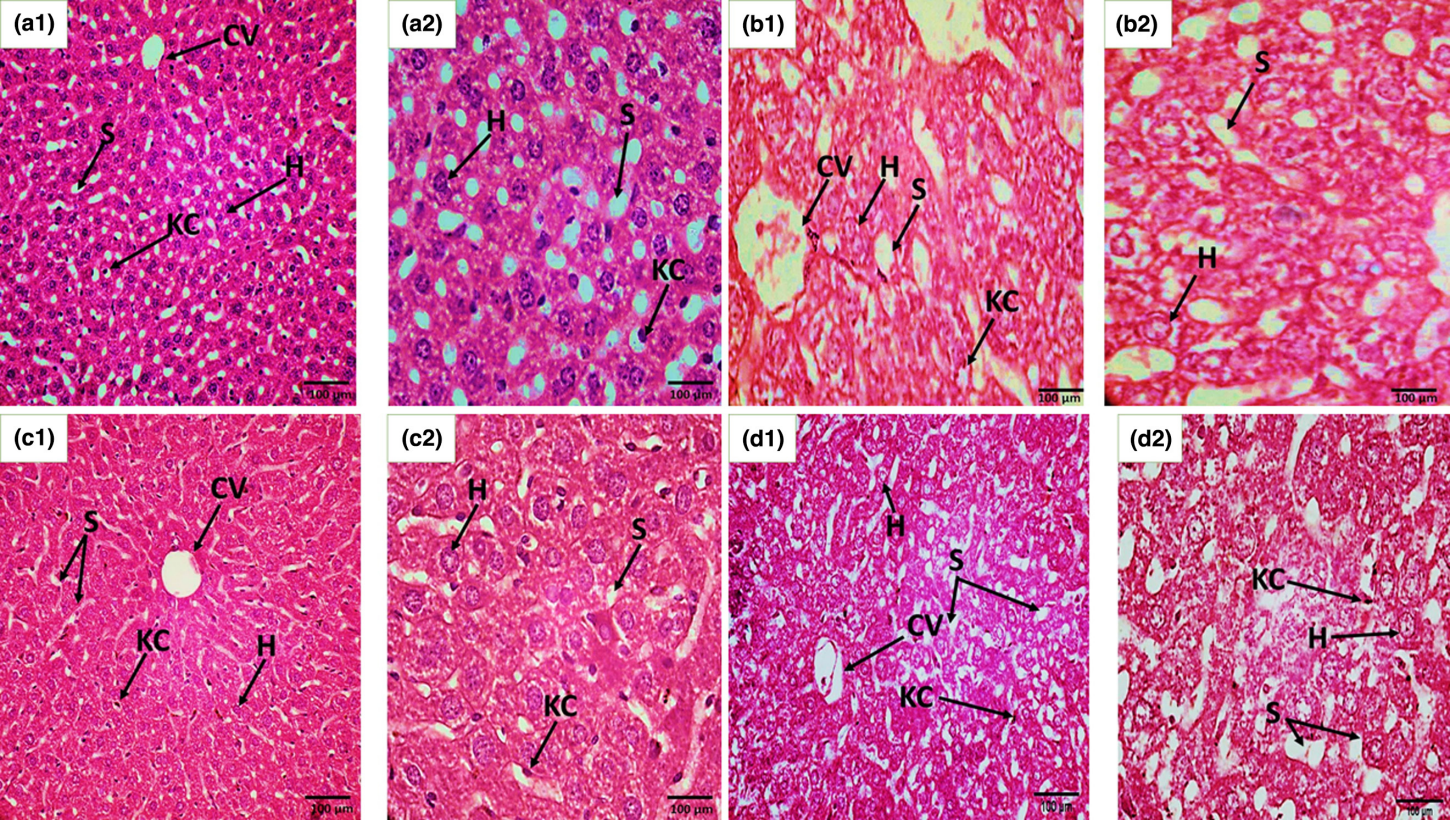
Histological sections of the liver tissues of F0 mothers (preparturition) recovered on the 18th day of GP. (a1 and a2) control group section shows the normal structure of hepatocytes (H), portal, sinusoidal spaces (S), central vein (CV), and Kupffer cell (KC), (b1 and b2) Dose: ZnO NPs exposed liver section shows the binucleated structure of hepatocytes (BNH), congestion of central vein (CV), dilated sinusoids (DS), and pyknotic nuclei in hepatocytes, (c1 and c2) Antidote: treated liver section shows the regular arrangement of Kupffer cells (KC), hepatocytes (H), and central vein (CV), and (d1 and d2) Dose + Antidote: treated liver section shows vacuolated hepatocytes (VH) and central vein (CV) disrupted epithelial layer (En), sinusoidal spaces (S), and seldom Pyknotic nuclei in Kupffer cells. H and E staining at 40× (a1, b1, c1, and d1) and at 100× (a2, b2, c2, and d2).

ZnO NPs treated F0 mothers and a few specimens of the dose + antidote group showed histopathological lesions in the liver sections eviscerated on the 18th day of the gestation period. Microanatomical observations indicated binucleated hepatocytes, central vein congestion, sinusoidal dilations, pyknotic nuclei, Kupffer cell activations, and liver steatosis as compared to the control group as shown in Figure 5.

#### 3.5.2 Microanatomy of maternal liver at postparturition

**FIGURE 6:**
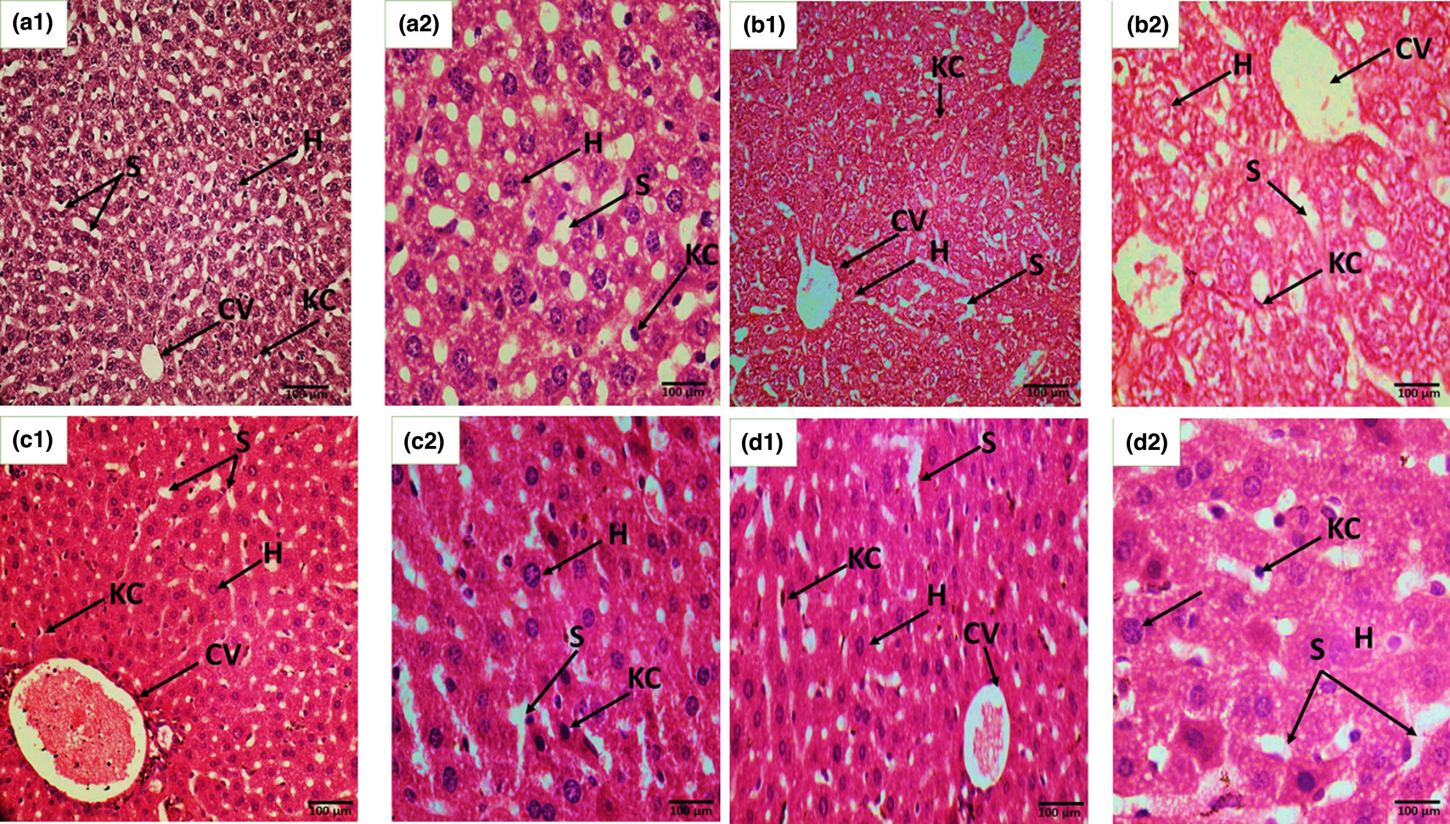
Histopathological sections of the liver tissues of F0 mother postparturition week 8. (a) Control section shows the normal structure of hepatocytes (h), portal vein (PV), sinusoidal spaces (s), central vein (cv), epithelial layer (EL), portal vein (PV); (b) Dose: ZnO NPs treated mother liver section shows sinusoidal dilation (SD) portal vein (PV), vacuolations (V), and the liver steatosis (LS); (c) Antidote: orange juice treated (antidote group) mother liver section shows well‐defined hepatocytes (H) central vein (CV), Kupffer cell, and sinusoidal spaces (S); (d) Dose + Antidote: sections of ZnO NPs and orange juice treated mother indicated little bit dilation of central vein (CV), pyknotic nuclei (pn), normal hepatocytes appearance (H), and sinusoidal spaces (s). H and E staining at 40× (a1, b1, c1, and d1) and at 100× (a2, b2, c2, and d2).

ZnO NPs intoxicated F0 mothers when dissected at postparturition week 8. Histopathological analysis indicated normal hepatocytes and central vein, but congested portal veins, sinusoidal dilations, and vacuolations were frequently observed probably due to the fat accumulation, as compared to control group as shown in Figure 6.

#### 3.5.3 Histopathological analysis of F1 pups' liver

Histopathological analysis of fetal liver in the control group on day 18 of gestation indicated a regular pattern of developing hepatocytes arranged in parallel arrays around the central vein, showing endothelial cells forming hepatic sinusoids uniformly in Figure 6a. In the dose group, distorted arrangement of developing hepatocytes with apoptotic zones, and necrotic endothelial cells resulting in malformed sinusoidal spaces with congested central veins can be observed in Figure 6b, whereas the dose + antidote group showed a deformed central vein as a consequence of epithelial degeneration that aligned the central vein, hepatic sinusoidal obliterations, and some more defects, these lesions are less obvious/severe than the NPs exposed group, which ensured protective efficacy of orange juice (Figure 6d). The liver sections of pups exposed to OJ through their mother during the prenatal period are almost similar to control groups, showing the normal formation of central vein, hepatocytes, Kupffer cells, and sinusoidal spaces as well (Figure 6c).

#### 3.5.4 Histopathological analysis of F1 offspring

**FIGURE 7:**
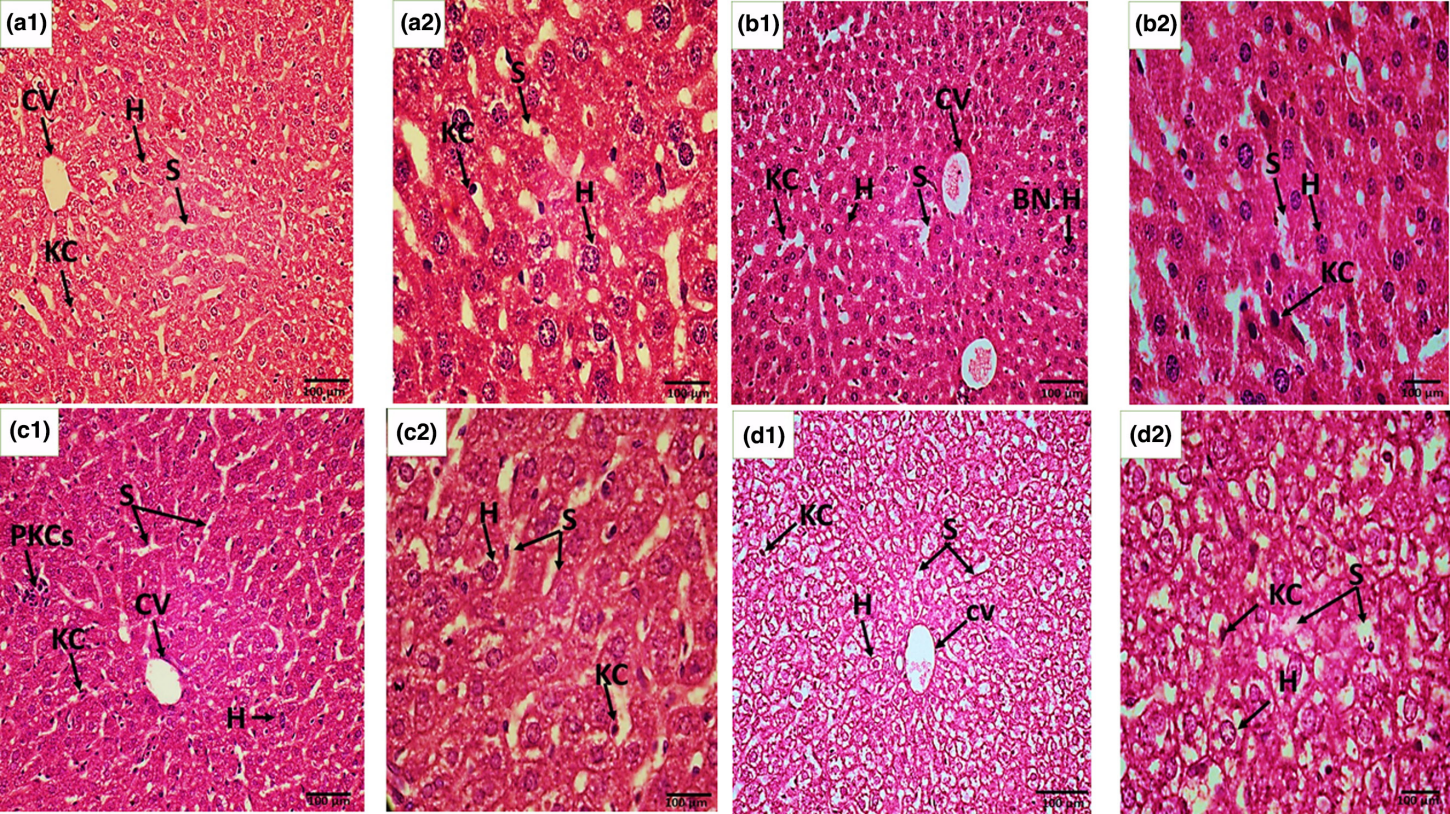
Histopathological sections of the liver tissues of F1 pups recovered on the 18th day of gestation after prenatal exposure to ZnO NPs through F0 mothers. (a) Control, developing central vein (DCV), sinusoids (s), hepatocytes (h); (b) Dose group shows necrotic endothelial cells (Ne), pyknosis (P), malformed hepatocytes (Hf), apoptotic zones (stars), and sinusoidal dilation (DS), congested central vein (CV); (c) Antidote shows central vein (CV), the epithelium (E), hepatic sinusoids (S), hepatocytes (H) and (d) Dose + antidote group: sinusoidal dilation (DS) and congested central vein (CV), amyloid fibril (Af). H and E staining at 40× (a1, b1, c1, and d1) and at 100× (a2, b2, c2, and d2).

**FIGURE 8:**
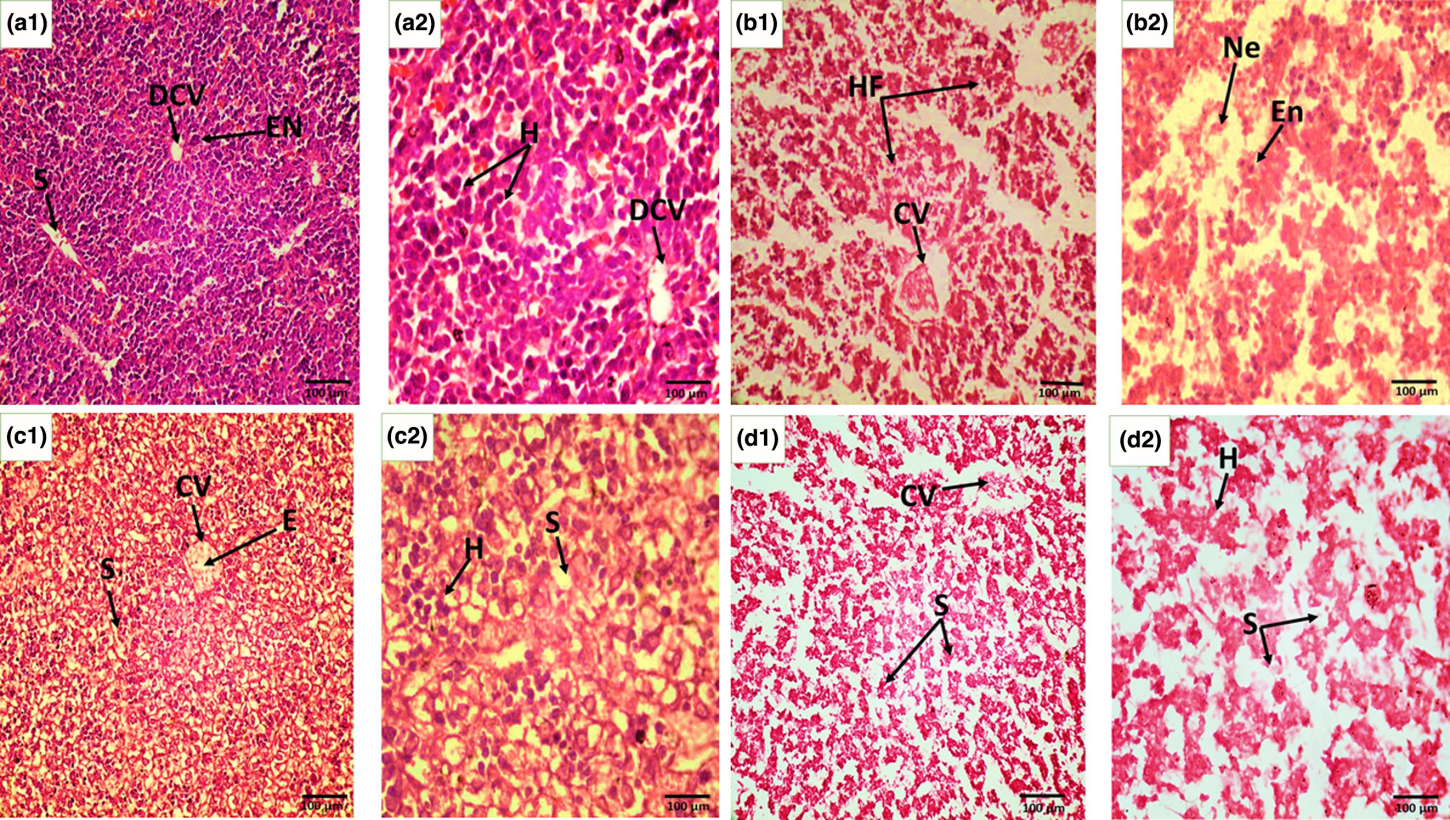
Histopathological sections of F1 offspring (8 weeks old) prenatally treated with ZnO NPs through F0 mothers. (a) The control section shows the normal structure of hepatocytes (h), portal vein (PV), sinusoidal spaces (S), central vein (cv), and epithelial layer (EL); (b) Dose group: F1 generation liver sections shows cytoplasmic vacuolization of hepatic cells (CVH), congested central vein (CCV) seldom excessive sinusoidal spaces (s), pyknosis (Pn), and binucleated hepatocytes (BN.H); (c) Antidote: orange juice treated mother; F1 mice liver section shows regular hepatocytes (H), Kupffer cell (KC), central vein (CV); (d) Dose + Antidote shows congested portal vein (CPV), regularly arranged hepatocytes (H), well‐developed central vein with congestion (CV), ballooned hepatocytes (bh). H and E staining at 40× (a1, b1, c1, and d1) and at 100× (a2, b2, c2, and d2).

Ultrastructure of the control group's liver in F1 mice in eighth week of the postnatal period showed normally located central vein, hepatocytes defined in regular parallel fashion around the central vein, and hepatic sinusoids wrapped with endothelial and Kupffer cells in control group, whereas dose and dose + antidote groups showed uniformed central vein with little bit congestion, binucleated, ballooned, and vacuolated hepatocytes. Pyknotic Kupffer cells and pyknotic nuclei in hepatocytes were also observed (Figure 7). The histological lesion in F1 offspring's liver can be seen but these are comparatively adulterated in the next generation as seen in their mothers' (F0) liver tissue, which is directly exposed. The liver features of F1 mice exposed to OJ via their mothers prenatally were comparable with the control group, showing well‐formed hepatocytes, central vein, and sinusoidal spaces, as shown in Figure 8.

### 3.6 Antioxidant status of liver

#### 3.6.1 Antioxidant capacity of maternal liver at preparturition

**FIGURE 9:**
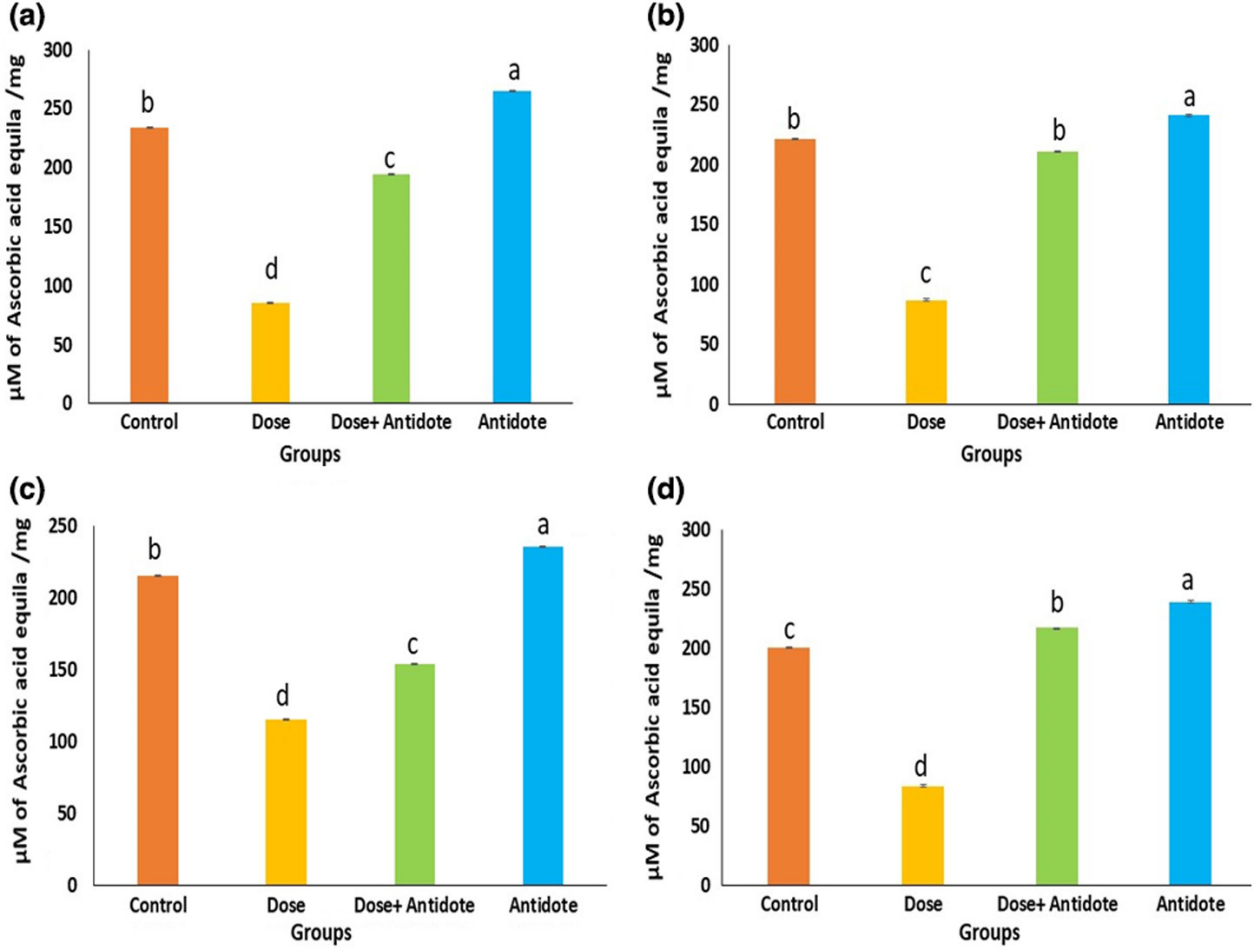
Graphs showing antioxidant potential within the liver tissue homogenates. (a) The antioxidant capacity of maternal liver at preparturition; (b) Antioxidant capacity in the liver of F1 pups on 18th day of gestational period; (c) Antioxidant capacity of maternal liver at postparturition; (d) Antioxidant capacity in F1 liver recovered in eighth week of postnatal period. Data represent in terms of the mean of FRAP values ± SEM. *p* < .05.

Ferric reducing antioxidant power assay of maternal liver homogenates at the 18th day of gestation showed a significant decrease (*p* < .01) in antioxidant activity (85 ± 0.34 Equiv μM ascorbic acid/g) of the livers exposed to NPs in comparison with control (234 ± 0.46 Equiv μM ascorbic acid/g). The antidote group (265 ± 0.65 Equiv μM ascorbic acid/g) showed a slightly improved antioxidant status of the liver against the control, whereas antioxidant activity notably reduced (*p* < .05) in the dose + antidote group (194 ± 0.25 Equiv μM ascorbic acid/g) as compared to control, but a detectable increase was observed when compared with the dose group (ZnO NPs) as shown in Figure 9a.

#### 3.6.2 Antioxidant capacity of the fetal liver (F1 pups)

Antioxidant status of 18‐day‐old fetal liver maternally exposed to NPs decreased significantly (87 ± 0.96 Equiv μM ascorbic acid/g) as compared to control at *p* < .05, while in other groups, antioxidant capacity of the fetal liver was comparable to the control, as shown in Figure 9b.

#### 3.6.3 Antioxidant capacity of maternal liver at postparturition week 8

Antioxidant activity of maternal liver at postparturition week 8 decreased significantly in the dose group (115 ± 0.34 Equiv μM ascorbic acid/g) relative to the control group (215 ± 0.46 Equiv μM ascorbic acid/g) analyzed through FRAP assay, but the effect is ameliorated with the time because FRAP values are increased from the maternal liver at preparturition. Antioxidant activity in dose + antidote group slightly decreased (154 ± 0.25 Equiv μM ascorbic acid/g), whereas the antidote group showed a remarkable increase in antioxidant activity (235 ± 0.65 Equiv μM ascorbic acid/g) compared to control (Figure 9c).

#### 3.6.4 Antioxidant status of F1 offspring's livers

At postnatal week 8, F1 offspring's liver was eviscerated to check their antioxidant capacity through FRAP assay. The livers of F1 mice that received intrauterine NPs exposure showed a significant decrease (84 ± 0.96 Equiv μM ascorbic acid/g) in antioxidant activity of the liver homogenates as compared to control even at postnatal week 8, while in antidote and dose + antidote groups, mice liver status was much improved (*p* < .05) in the next generation, probably due to the OJ protection, which was assayed by using the FRAP method as shown in Figure 9d.

### 3.7 Total phenolic content

**FIGURE 10:**
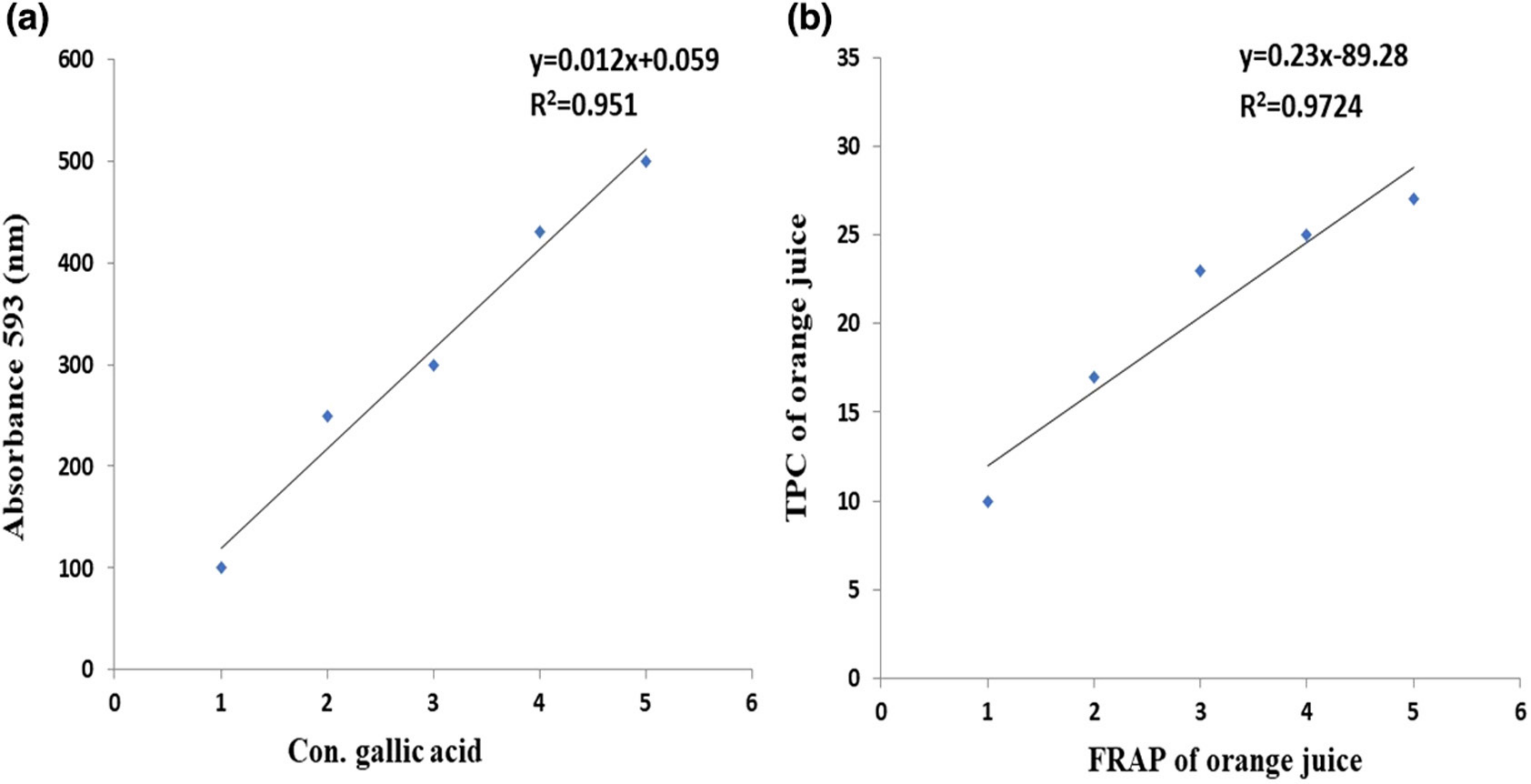
(a) Plotted regression curve shows the concentration of gallic acid versus absorbance for the determination of phenolic acid. (b) Plotted regression curve shows the strong correlation between TPC and FRAP values of orange.

Mean FRAP and TPC values of fresh orange juice were 288.5 ± 0.20 Equiv. μM ascorbic acid/100 mL and 4.211 ± 0.09 equiv. mg, Gallic Acid/100 mL, respectively, which showed a strong interrelationship between antioxidant potential and total phenolic content of fresh orange juice as shown in Figure 10.

## 4 DISCUSSION

For the time being, trace metals and metal oxide nanomaterials became multifunctional materials, which ensure their recurrent exposure to humans. So, current study explored trans‐placental and multigenerational embryotoxicity and hepatotoxicity after oral exposure to ZnO nanoparticles and the protective potential of fresh orange juice as well. Mice were selected as in vivo models for the manifestation of the experiment due to their similarities with human metabolic, biochemical, and physiological mechanisms. Toxic effects of ZnO NPs were investigated in F0 mothers, F1 pups during the prenatal period, and F1 offspring at the eighth week of the postnatal period. As ZnO NPs are extensively used in food packaging, ceramics, and cosmetics owing to their admirable and specific antiseptic and photosensitive features (Hoseinnejad et al., 2018), it may gain direct entry into our body so an oral pathway was selected for exposure to investigate its toxicological properties.

Reproductive and hepatic toxicity has been identified as a salient part of the overall toxicological investigations. The organogenetic period is the most crucial time in pregnancy. Our results showed that intrauterine exposure to nanoparticles caused various developmental defects like limb defects, open eyelids, short tails, and hemorrhage in 18‐day‐old embryos. Our findings are supported by Yan et al., who reported due to their nano size, ZnO nanoparticles cross the placental barriers easily. These particles accumulate in female reproductive organs, which may lead to developmental anomalies in the fetuses (Chen et al., 2020; Choi et al., 2016; Yan et al., 2020). Another study reported embryological imperfections in hens too (Zhao, Li, et al., 2016; Zhao, Ren, et al., 2016).

Pregnant females (dissected at pre‐ and postparturition) and F1 mice showed a decrease in body weight gain and increase in liver weight on oral exposure to ZnO nanoparticles in comparison with the control and OJ‐treated group. Few aspects of our findings were close to the finding of Yousef et al., 2019, they reported that ZnO NPs alone and in combination with AlO NPs resulted in decreased body weight gain in rats against control and AlO nanoparticles as well, but here organ weight is also decreased in the same manner contrary to our findings. As ZnO nanoparticles can easily cross intestinal and placental barriers, so they accumulate in the maternal and fetal liver tissues, respectively, which may lead to retardation of maternal along with fetal growth and development. Comparing the toxic effects of both ZnO nanoparticles and ZnSO_4_ given in the same concentration revealed that the ZnO nanoparticles produce more hepatotoxicity, which causes a decrease in maternal body weight and an increase in liver weight. Some studies reported ZnO nanoparticles to cause oxidative distress in tissues which presumably is the reason for toxicity (Jo et al., 2013; Lee et al., 2016; Sakr & Steenkamp, 2021). Increased liver weight in NPs exposed group might be due to the inflammation or functional compensation.

Our study revealed that the liver key regulators, ALT, AST, and ALP levels in serum increased significantly in the dose group as compared to control at day 18 of the gestational period, which indicates a hepatic malfunction, whereas in the dose + antidote group, enzyme level increased but less than dose group. Total bilirubin levels showed a significant decrease in the dose group, while there was no notable difference in dose + antidote group and antidote‐treated group as compared to control. When these enzymes were analyzed in the next generation at postnatal week 8 and in mothers at postparturition week 8, a remarkable increase was observed in NPs exposed mothers and offspring against control but less severe than previous results. It means with time NPs' effect is diluted to some extent. Other biochemical markers showed that total protein was higher, whereas the A/G ratio was lower in NPs exposed mothers and fetuses, which is usually a sign of inflammation or liver malfunction. These results also justify our biometric findings that increase in the liver weight is probably due to inflammation. Many other types of research also support our results, the deviation of the liver function markers after ZnO NPs exposure (Filippi et al., 2015; Mansouri et al., 2015; Wang et al., 2017; Yan et al., 2012).

After biochemical assessments, hepatotoxicity was further confirmed through histopathological analysis. ZnO NPs exposed mothers, F1 pups, and F1 offspring showed malformed anatomical structures of hepatocytes, congested central vein, sinusoidal dilations, pyknotic nuclei, and activated Kupffer cells in comparison with control (Hao et al., 2017). These hepatic lesions were less obvious in the group, which is coadministered with OJ. Secondly, histopathological defects were decreased in order F0 mothers at preparturition > F1 pups > F0 mothers at postparturition > F1 offspring. In F1 offspring at postnatal week 8, anatomical lesions were less pronounced as compared to mothers and F1 pups' mean NPs effect diluted with age. Biodistribution studies show that the spleen, kidney, and liver are the main target organs for fabricated NPs when taken orally (Lee et al., 2016). Our histopathological findings are in line with the studies reported by Almansour et al. (2017) that long‐term exposure to ZnO nanoparticles can lead to liver dysfunction, which may cause liver injuries in rodents. But supporting data for F1 pups and F1 offspring hepatotoxicity are missing to relate to our results.

Among various mechanisms like genotoxicity, lipid peroxidation, and inflammatory pathways triggering involved in ZnO NPs induced toxicity, overproduction of ROS is the most common one. In our study, we check the antioxidant status of the liver in the liver homogenates through FRAP assay. Higher FRAP values in control and OJ exposed livers showed greater antioxidant capacity in the livers of the aforementioned groups; conversely, ZnO NPs exposed livers (directly or indirectly) showed the poor antioxidant status of the liver against control. But NPs‐OJ cotreated group showed improvement in the liver's antioxidant power to a great extent.

Many reports illustrated that the protective potential of orange juice was due to the presence of phytochemicals polyphenols and Vitamin C, which acts as an antioxidant, increases the level of iron in the liver, and reduces oxidative stress caused by ROS as reported by Hallfrisch et al. (1994), Youness et al. (2016), and Song et al. (2017). Antioxidant properties of fresh orange juice are evaluated through FRAP and TPC assays, which showed strong correlations between antioxidant activity and TPC of orange juice, a similar result reported by Stella et al. (2011).

## 5 CONCLUSION

Conclusively, the current research showed induced teratogenicity and multigenerational hepatotoxicity after ZnO nanoparticle's direct exposure to mothers and indirect exposure (intrauterine) to offspring during their organogenetic period. Overall, biometric parameters of the dose group indicate a decrease in body weight gain, both in mothers and F1 offspring, whereas increased liver weight in both cases, presumably due to inflammation, later on abnormally high serum proteins and A/G ratio confirmed the abovementioned results. Birth defects were frequently observed in 18‐day‐old fetuses in the ZnO nanoparticles treated group contrary to other groups, only mild hemorrhage and limb extension can be seen in the cotreated group. Enzymatic assays of F0 mothers and F1 offspring showed elevated levels of the liver functional markers, ALT, AST, and ALP in NPs exposed groups against all other groups, these deviations ameliorated with OJ and passage of time to a great extent. Histopathological lesions were well observed in NPs exposed livers of mothers (preparturition), and F1 pups, but less pronounced in the group of the same cadre, which was coadministered with OJ and NPs. Anatomical defects of the livers in 8‐week‐old F1 offspring and mothers (postparturition week 8) were restored with time considerably. Further antioxidant properties of fresh orange juice are proved through FRAP and TPC assays, high phenolic content and FRAP values were correlated significantly, which in all probability played a role to reduce oxidative stress‐induced embryotoxicity and hepatotoxicity as well. So, fresh orange juice is recommended for pregnant females to get protection against the unseen effects of ZnO nanoparticles, particularly during the organogenetic period.

## AUTHOR CONTRIBUTIONS

**Chaman Ara:** Conceptualization (equal); investigation (equal); methodology (equal); resources (equal); software (equal); writing – original draft (equal). **Shagufta Andleeb:** Formal analysis (equal); methodology (equal); software (equal); supervision (equal); writing – original draft (equal). Methodology (equal); supervision (equal); validation (equal); writing – original draft (equal). **Shaukat Ali:** Conceptualization (equal); investigation (equal); methodology (equal); supervision (equal). **Barirah Majeed:** Conceptualization (equal); data curation (equal); formal analysis (equal); investigation (equal); methodology (equal). **Asia Iqbal:** Formal analysis (equal); investigation (equal); software (equal); writing – original draft (equal); writing – review and editing (equal). **Aliza Muzamil:** Conceptualization (equal); formal analysis (equal); funding acquisition (equal); investigation (equal); methodology (equal). **Madeeha Arshad:** Conceptualization (equal); formal analysis (equal); funding acquisition (equal); investigation (equal); methodology (equal). **Asma Chaudhary:** Funding acquisition (equal); resources (equal); ); writing – original draft (equal); writing – review and editing (equal).

## CONFLICT OF INTEREST STATEMENT

All authors declare that there are no conflicts of interest.

## Data Availability

The data that support the findings of this study are available from the corresponding author upon reasonable request.
